# ASAP: an environment for automated preprocessing of sequencing data

**DOI:** 10.1186/1756-0500-6-5

**Published:** 2013-01-04

**Authors:** Eric S Torstenson, Bingshan Li, Chun Li

**Affiliations:** 1 Center for Human Genetics Research, Vanderbilt University, Nashville, USA; 2 Department of Molecular Physiology and Biophysics, Vanderbilt University, Nashville, USA; 3 Department of Biostatistics, Vanderbilt University, Nashville, USA; 4 Center for Human Genetics Research, Vanderbilt University Medical Center, 519 Light Hall, Nashville, TN, 37212-0700, USA

**Keywords:** Next-generation sequencing, Data processing, Automation, Computer cluster

## Abstract

**Background:**

Next-generation sequencing (NGS) has yielded an unprecedented amount of data for genetics research. It is a daunting task to process the data from raw sequence reads to variant calls and manually processing this data can significantly delay downstream analysis and increase the possibility for human error. The research community has produced tools to properly prepare sequence data for analysis and established guidelines on how to apply those tools to achieve the best results, however, existing pipeline programs to automate the process through its entirety are either inaccessible to investigators, or web-based and require a certain amount of administrative expertise to set up.

**Findings:**

Advanced Sequence Automated Pipeline (ASAP) was developed to provide a framework for automating the translation of sequencing data into annotated variant calls with the goal of minimizing user involvement without the need for dedicated hardware or administrative rights. ASAP works both on computer clusters and on standalone machines with minimal human involvement and maintains high data integrity, while allowing complete control over the configuration of its component programs. It offers an easy-to-use interface for submitting and tracking jobs as well as resuming failed jobs. It also provides tools for quality checking and for dividing jobs into pieces for maximum throughput.

**Conclusions:**

ASAP provides an environment for building an automated pipeline for NGS data preprocessing. This environment is flexible for use and future development. It is freely available at http://biostat.mc.vanderbilt.edu/ASAP.

## Background

Modern sequencing technologies have greatly improved our capability of acquiring deep sequencing data on a large scale and in a timely fashion. However, the large amount of data presents many new challenges to researchers, including a significant amount of time and effort on preprocessing raw sequencing reads into variant calls that are ready for statistical analyses. This process involves multiple steps and several independent programs. For example, for species with a reference genome available, sequence reads are often initially aligned to the reference genome using a mapping program such as BWA (Li & Durbin [1]). Additionally, reads aligned to insertion-deletion regions may require local realignment to minimize false variant calls, and base quality scores may require recalibration to reflect empirical error rates; these can be achieved with GATK (McKenna et al. [2]). Moreover, variant calls may require filtering for false call removal and annotation for downstream analyses. The various steps require different programs and there may be multiple programs available for some steps; for example, variants can be called using GATK, samtools (Li et al. [3]), or UMAKE (Kang et al. [4]). In some of the steps (e.g., local realignment and variant calling), it may be desirable to process multiple samples together to borrow information across samples. Most steps can also benefit from distribution of jobs on multiple computer nodes. Managing these tasks manually on hundreds of samples is a daunting process and is error prone. It is therefore desirable to have a pipeline to automate these tasks, and it is even better to have a flexible environment for building an automated pipeline to allow adding or removing steps and using alternative programs.

Computer clusters have become available in recent years, granting researchers access to hundreds and sometimes thousands of processors at affordable prices, yet few if any of the applications required for sequence data preprocessing currently support distribution of tasks over a computer cluster. In addition, few researchers have administrative access to their clusters. Galaxy (Goecks et al. [5]), a general purpose bioinformatics tool, can be used to help resolve some of these issues. However, it requires a certain amount of administrative expertise to get started as well as a significant amount of resources dedicated to host and maintain the Galaxy server. Some sequencing facilities may have pipelines for internal use; however, these internal pipelines may not be accessible to investigators who wish to have a full control over data preprocessing.

We designed Advanced Sequence Automated Pipeline (ASAP), an object-oriented application framework, to resolve the issues above while minimizing human involvement associated with processing a large volume of sequencing data so that researchers can quickly start their statistical analyses as well as incrementally add new data as is often necessary for large-scale research projects. ASAP maximizes local cluster usage with minimal user experience and no administrative access. It offers an easy-to-use command interface for submitting and tracking jobs as well as resuming failed jobs. It also provides tools for checking data quality. The current version contains some of the component programs most commonly used for preprocessing human sequence data, which can be replaced with alternative tools as needed. ASAP processing scripts can also be run in serial for those who want a pipeline for processing their data but have no access to a computer cluster.

## Implementation

We developed ASAP as an environment to streamline preprocessing of next-generation sequencing data from raw sequence reads to annotated variant calls. It was designed to do the following: 1) produce scripts for data processing according to the user’s pipeline configuration, 2) maximize hardware usage, 3) manage jobs, 4) ensure data integrity, and 5) minimize user involvement while maximizing flexibility for use and future development.

### Processing capabilities and configuration

**Figure 1:**
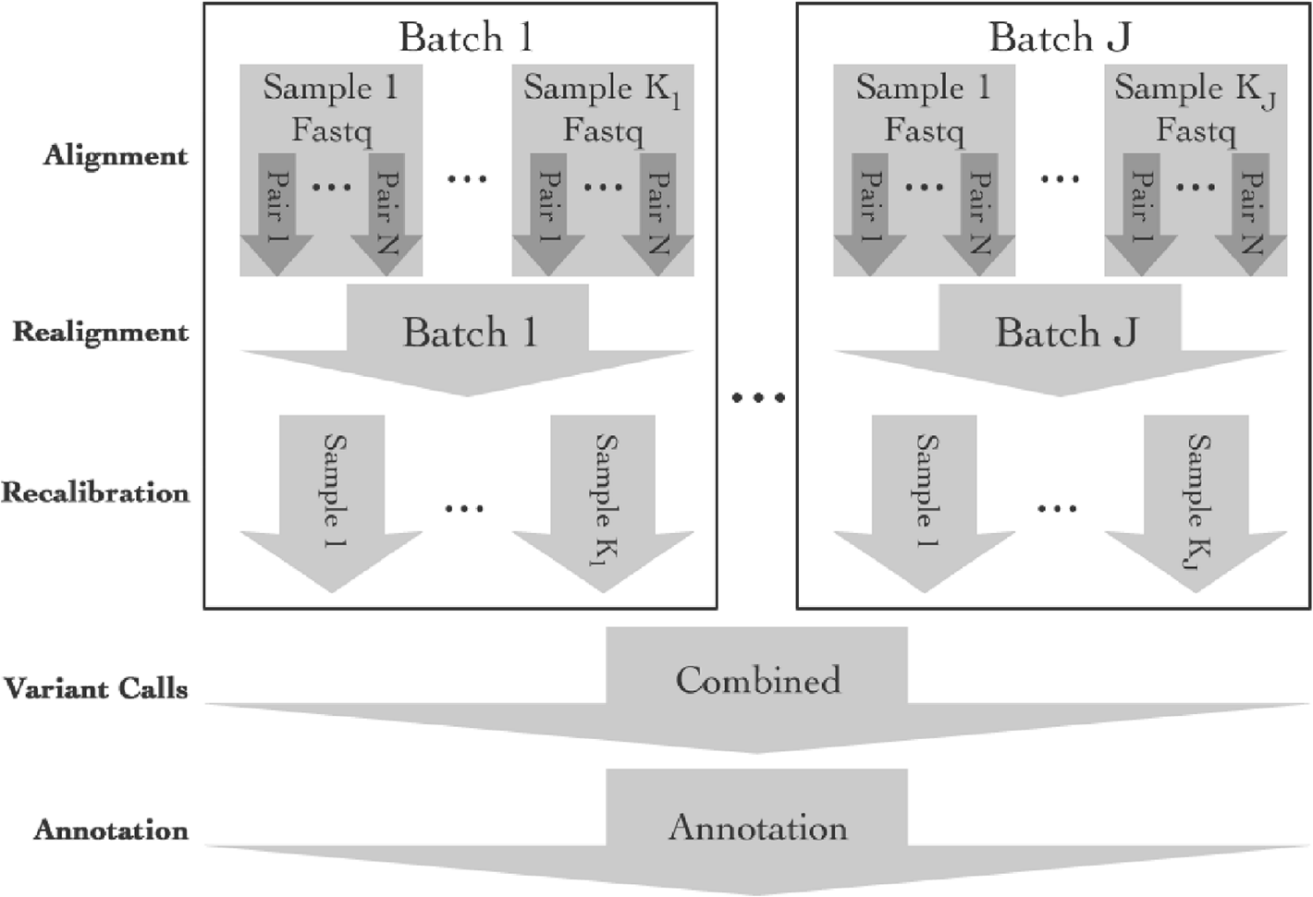
**Diagram of the major steps of ASAP. **All the steps are optional and the grouping of samples is flexible.

ASAP can currently execute five major steps using established tools in sequence data preprocessing (Figure 1): 1) alignment of sequence reads to a reference genome, 2) local realignment around insertion-deletion regions, 3) quality score recalibration, 4) variant calling (for SNPs and insertions/deletions), and 5) annotation of variants. Users define their preprocessing pipeline by selecting appropriate steps and import data either as fastq for alignment, or as bam files at any point within the pipeline. By importing bam files, users can integrate external data with data processed by ASAP without having to tweak scripts to merge the data. ASAP currently provides alignment to a reference sequence with BWA, variant calling using GATK, samtools, and UMAKE, and annotation using ANNOVAR (Wang et al. [6]). A user can customize ASAP to use other programs and even add new processing steps to allow the construction of highly customized pipelines. In some of the steps (e.g., local realignment and variant calling), it may be desirable to process multiple samples together to borrow information across samples. ASAP provides various sample grouping schemes during these steps (Figure 1) to give researchers maximum flexibility on how the sample are pooled at different steps.

ASAP uses a single configuration file for the whole process. It allows users to easily create baseline configurations and can incorporate parameters from multiple sources. This can help minimize setup time and take advantage of template configurations shared as “best practices” by the research community. These template configuration files can be partial, stripped of any sensitive or study-specific parameters, and ASAP will use its default values for all unspecified parameters. To achieve reproducibility, all settings are stored in this one file, which can be edited with any text editor and all processing scripts are retained for future inspection.

Users can also specify directory paths for permanent and temporary data. The permanent directory contains output and error logs, the job scripts, and the final bam and variant calls. The temporary directory contains intermediate files that are required by subsequent steps and may ultimately be discarded by users.

### Hardware maximization

Next-generation sequencing can generate a large amount of data. For example, human exome sequencing data can consume over 5 gigabytes (GB) at ≥30x depth of coverage after file compression, and whole-genome sequencing at the same depth can exceed 200 GB in size. Processing files of this size in a serial fashion is time consuming and is unnecessary. ASAP can improve the speed of data processing by providing tools for dividing tasks to allow parallel processing and by using threads when they are supported by component programs, such as BWA and GATK. There are three ways for dividing tasks: 1) split fastq files for parallel alignment, as illustrated in Figure 1, 2) execute jobs by chromosome whenever possible, and 3) optionally split chromosomes into smaller segments for variant calling. As chromosomes have different sizes, ASAP also allows flexible grouping of chromosomes to balance the time spent on the jobs. By splitting the data up in this manner and distributing the jobs to multiple nodes, these optimizations can provide a significant boost in throughput with no negative impact on the results.

When processing large files, input and output bandwidth becomes vital for execution speed. Network traffic can be heavy even for a dedicated storage space of a computer cluster. To minimize network traffic and further improve processing, ASAP makes extensive use of Unix pipes eliminating the overhead associated with writing and reading temporary files. When ASAP must use intermediate files, it offers users the ability to prioritize alternate storage for these files, allowing them to take advantage of local storage when it is available.

### Job management

ASAP doesn’t process data directly. Instead, based on the user’s pipeline configuration, it produces short Ruby scripts and can submit them to a computer cluster. These scripts are straightforward, contain explicit calls to component programs, and are readable by anyone with basic knowledge of programming syntax. By default new scripts do not overwrite previous scripts, thus leaving a complete record of what was done. When used on a computer cluster, ASAP correctly manages job submissions and dependencies, eliminating delays between the steps of a user’s pipeline.

A job can fail due to various reasons, and having many jobs running increases the difficulty of job tracking. The scripts generated by ASAP use tokens to identify their execution status while running under ASAP job management. This built-in tracking functionality helps reduce delays in processing by allowing the user to rerun ASAP to generate only those jobs that have failed along with their dependencies and resubmit them even while the other jobs are still properly queued or actively running. ASAP also provides simple commands to display job status and log contents. When adding new samples to a preexisting project, ASAP will correctly process the new data independent of the data already processed, rerunning previously completed steps only when necessary based on the pipeline defined by the user.

### Data integrity and quality control

Data integrity is very important in scientific research. ASAP provides two mechanisms to ensure the jobs are complete. The scripts intercept error codes passed from component programs and halt execution upon receiving an error. They also stop with an error if there is insufficient disk space when moving and copying data.

ASAP also provides summaries that are useful for quality checking. For example, it calculates transition-transversion ratio and het/hom ratio (defined as the number of mutations detected as heterozygous divided by the number of homozygous mutations). These numbers allow the investigator to quickly recognize problems such as contamination or processing errors. Other summaries such as those from “samtools flagstat” will be generated as well. To further assist in the preparation for analysis, ASAP also provides the option of annotating the variant calls using ANNOVAR.

### Flexible design for use and development

Because the current state of sequence data processing is rapidly changing, ASAP was designed to be flexible for use and development. One design element that allows such flexibility is its extensive reliance on a plug-in architecture. Plug-ins are found by performing “live searches” for ASAP components such as steps, templates, and configuration classes. This allows developers to follow simple naming conventions and class inheritance to add new functionality to ASAP without having to modify any of the preexisting code. Users can add new components, for example, to use an alternative aligner or variant caller, and they can add entirely new steps to extend a pipeline in different ways. Users can also add a new execution mechanism to modify the manner in which scripts are launched, for example, to adapt ASAP to another cluster platform. Development of new functionalities requires only a moderate amount of object-oriented programming technique and knowledge of Embedded Ruby (ERB).

### Minimize user involvement

ASAP can be installed without administrative rights and requires only Ruby 1.9.3 and its SQLite3 gem, which are often available on Linux distributions or can easily be installed with or without administrative rights. For users who haven’t already downloaded the necessary component programs, ASAP can be used to download and install those tools automatically, and will record the newly installed software for use in all future runs. Once system preparation is complete, starting the whole data preprocessing pipeline can require as few as three commands, depending on the organization of the data to be processed. During the processing, ASAP offers a simple interface for checking status and restarting failed jobs.

## Results

We ran ASAP on 5 human exome and 2 whole-genome sequencing data on the computer cluster at Vanderbilt University, which runs 64-bit Linux on around 4,000 processor cores each with 3–16 GB of memory and uses Portable Batch System (PBS) for resource management. All data were paired-end 100nt reads with >30x coverage. No user intervention was needed after the initial setup. We used 2 threads for the steps wherever threading was supported. It took <1 day to finish the exomes and <2 days for the whole genomes, running through initial alignment, local realignment, quality score recalibration, variant calling, and annotation.

Table 1 contains the number of jobs, average time, and maximum memory usage for jobs of each of the major steps. The run time was much shorter than running the tasks in serial. For example, the initial alignment finished in less than 44 minutes for all 5 exomes and less than 5 hours for the whole genomes. The maximum memory usage varied across the steps, but all were less than 5 GB per job. For all the steps except initial alignment, the memory requirement may grow as the number of samples increases but it can also be reduced by either increasing the number of jobs or lowering the number of threads.

**Table 1:** Number of jobs, average run time (in minutes), and maximum memory used (in GB) for the major steps of preprocessing

	**5 exomes**			**2 whole genomes**		
	**# jobs**	**Average time**	**Maximum memory**	**# jobs**	**Average time**	**Maximum memory**
Alignment	38	43.6	4.3	82	277.5	4.5
Realignment	18	29.0	3.5	36	181.7	4.8
Recalibration	80	7.0	3.3	32	109.3	2.9
SNP calling	136	1.6	3.2	136	9.8	3.1
Annotation	24	28.6	2.7	24	29.7	2.7
Total	311			316		

## Discussion

We have developed an environment for building a pipeline to preprocess NGS data and for managing its jobs. The pipeline scripts will automate the steps in sequence data preprocessing from raw sequence reads to annotated variant calls as defined by the user. Such a pipeline not only provides automated processing, but also ensures consistency, reproducibility, maximization of hardware usage, use of “best practice” procedures and settings, and job traceability. Failing to properly cover any of these issues can result in significant delay in starting statistical analyses. ASAP is not intended to be a replacement of existing component programs. The component programs in the current version are commonly used in practice today, and can be replaced with alternatives if necessary.

Sequencing data processing involves several steps, each having multiple parameters to set. Even if a sequencing facility offers data processing and variant calling, it may still be desirable to reprocess the data. For example, the settings used by a sequencing facility may not be appropriate for the purpose of a study; a study may have data generated at multiple facilities with different processing settings. ASAP is a timely tool for automatic processing of sequencing data while giving investigators full control of parameters. It can also be used by sequencing facilities.

ASAP is an open-ended framework, which can be extended to do many things. The current version provides features that are useful for processing DNA sequencing data for species with a reference genome available. RNA sequencing data often require different steps and programs, such as TopHat (Trapnell et al. [7]) for alignment and Cufflinks (Trapnell et al. [8]) for extracting expression information and performing statistical analyses. For species without a reference genome, NGS data often require *de novo* assembly and thus a different set of tools. These capabilities can be added to ASAP as plug-ins, and we plan to add these features in the future.

ASAP is also designed to take advantage of local clusters such as those available at many research institutions today. Specifically, the current version provides compatibility with Torque/PBS and Sun Grid Engine (SGE) based clusters. Support for other systems can be added if there is sufficient interest. For users who lack access to a computer cluster, ASAP can generate a collection of Ruby scripts and assist in running those scripts in a serial fashion. In addition, ASAP provides easy installation of component programs and a primer with an example dataset to help the user to get started quickly.

As cloud computing becomes common, there will be an interest in moving large-scale computation onto cloud clusters. At this point, it is still more expensive than using local clusters readily available to many investigators. ASAP can be modified to run under cloud platforms and we plan to work on that.

## Findings

ASAP is a user-friendly tool that can be used to automate script generation and job management for preprocessing sequencing data from raw reads to annotated variant calls. It also is highly configurable and extendable to fit a user’s need. The resulting output provides the user with the consistency and reliability required for sound analysis downstream. ASAP gives investigators full control of settings in all the processing steps with complete reproducibility. It maximizes hardware usage on a computer cluster while minimizing user involvement, and provides a flexible framework for use and future development.

## Availability and requirements

Project name: Advanced Sequence Automated Pipeline (ASAP)

Project home page: http://biostat.mc.vanderbilt.edu/ASAP

Operating systems: Linux/Unix, MacOS

Programming language: Ruby 1.9.3 or above

Other requirements: Ruby gem for SQLite3

License: GNU GPL

Any restrictions to use by non-academics: None

## Competing interests

The authors declare that they have no competing interests.

## Authors’ contributions

CL designed the study. EST implemented the program. BL provided guidance on program design and implementation. EST and CL drafted the manuscript. All authors read and approved the final manuscript.

## References

[B1] Li H Durbin R Fast and accurate short read alignment with Burrows-Wheeler transform Bioinformatics 2009 25 1754 1760 10.1093/bioinformatics/btp324 19451168 PMC2705234

[B2] McKenna A Hanna M Banks E Sivachenko A Cibulskis K Kernytsky A Garimella K Altshuler D Gabriel S Daly M DePristo MA The genome analysis toolkit: a MapReduce framework for analyzing next-generation DNA sequencing data Genome Res 2010 20 1297 1303 10.1101/gr.107524.110 20644199 PMC2928508

[B3] Li H Handsaker B Wysoker A Fennell T Ruan J Homer N Marth G Abecasis G Durbin R 1000 Genome Project Data Processing Subgroup The sequence alignment/map format and SAMtools Bioinformatics 2009 25 2078 2079 10.1093/bioinformatics/btp352 19505943 PMC2723002

[B4] Kang HM Jun G Sidore C Li Y Anderson P Trost MK Chen W Blackwell T Abecasis G UMAKE 2012 http://genome.sph.umich.edu/wiki/UMAKE.

[B5] Goecks J Nekrutenko A Taylor J Galaxy Team Galaxy: a comprehensive approach for supporting accessible, reproducible, and transparent computational research in the life sciences Genome Biol 2010 11 R86 10.1186/gb-2010-11-8-r86 20738864 PMC2945788

[B6] Wang K Li M Hakonarson H ANNOVAR: functional annotation of genetic variants from high-throughput sequencing data Nucleic Acids Res 2010 38 e164 10.1093/nar/gkq603 20601685 PMC2938201

[B7] Trapnell C Pachter L Salzberg SL TopHat: discovering splice junctions with RNA-Seq Bioinformatics 2009 25 1105 1111 10.1093/bioinformatics/btp120 19289445 PMC2672628

[B8] Trapnell C Williams BA Pertea G Mortazavi A Kwan G van Baren MJ Salzberg SL Wold BJ Pachter L Transcript assembly and quantification by RNA-Seq reveals unannotated transcripts and isoform switching during cell differentiation Nat Biotechnol 2010 28 511 515 10.1038/nbt.1621 20436464 PMC3146043

